# *Bupleurum* Polysaccharides Attenuates Lipopolysaccharide-Induced Inflammation via Modulating Toll-Like Receptor 4 Signaling 

**DOI:** 10.1371/journal.pone.0078051

**Published:** 2013-10-22

**Authors:** Jian Wu, Yun-Yi Zhang, Li Guo, Hong Li, Dao-Feng Chen

**Affiliations:** 1 Department of Pharmacology, School of Pharmacy, Fudan University, Shanghai, China; 2 Department of Pharmacognosy, School of Pharmacy, Fudan University, Shanghai, China; University of Hawaii, United States of America

## Abstract

**Background:**

*Bupleurum* polysaccharides (BPs), isolated from *Bupleurum smithii* var. *parvifolium*, possesses immunomodulatory activity, particularly on inflammation. Bacterial endotoxin lipopolysaccharide (LPS) triggers innate immune responses through Toll-like receptor 4 (TLR4) on host cell membrane. The present study was performed to evaluate whether the therapeutic efficacy of BPs on suppression of LPS’s pathogenecity could be associated with the modulating of TLR4 signaling pathway.

**Methodology/Principal Findings:**

LPS stimulated expression and activation of factors in the TLR4 signaling system, including TLR4, CD14, IRAK4, TRAF6, NF-κB, and JNK, determined using immunocytochemical and/or Western blot assays. BPs significantly inhibited these effects of LPS. LPS increased pro-inflammatory cytokines (TNF-α, IL-6, IL-1β, IL-12p40, and IFN-β) and NO production, evaluated using ELISA and Griess reaction assays, respectively. BPs antagonized these effects of LPS. Interestingly, BPs alone augmented secretion of some pro-inflammatory cytokines of non-LPS stimulated macrophages and enhanced phagocytic activity towards fluorescent *E.coli* bioparticles. In a rat model of acute lung injury (ALI) with pulmonary hemorrhage and inflammation, BPs ameliorated lung injuries and suppressed TLR4 expression.

**Significance:**

The therapeutic properties of BPs in alleviating inflammatory diseases could be attributed to its inhibitory effect on LPS-mediated TLR4 signaling.

## Introduction

Lipopolysaccharide (LPS), a major cell wall component of Gram-negative bacteria, is responsible for the overwhelming innate immune response of sepsis syndrome leading to multiple organ failure and death in septic patients [1]. As a potent microbial initiators of inflammation, LPS is regarded as a main factor of pneumonia resulting in acute lung injury (ALI), and its severe form, acute respiratory distress syndrome (ARDS), which is a leading cause of mortality in humans [2]. The host defense responses to LPS include production of a wide range of pro-inflammatory cytokines such as TNF-α, IL-6, IFN-β, as well as inducible NO synthase (iNOS) [3]. Although various factors and molecular activities are involved in these responses, LPS recognition by the host cells has been considered as a critical step to initiate inflammatory process. Thus, targeting this event may be a promising strategy for therapeutic intervention. 

Toll-like receptors (TLRs) play critical roles in host defense by sensing microbial pathogens and initiating innate and adaptive immune responses [4]. Among TLRs, TLR4 recognizes LPS and transmits its signals to immune sentinel cells, such as macrophages [4]. CD14, which is expressed as a glycosylphosphatidylinositol-linked protein within the plasma membrane of cells, serves as a co-receptor for LPS [5]. CD14 lacks a transmembrane domain and the binding of LPS to it triggers signal transduction through TLR4. TLR4 recruits and activates downstream signaling molecules, including myeloid differentiation primary-response protein 88 (MyD88), IL-1 receptor-associated kinase 4 (IRAK4), and TNF receptor-associated factor 6 (TRAF6) to initiate a cytoplasmic signaling cascade [4]. This signal transduction leads to activation of mitogen-activated protein kinases (MAPKs) [4], phosphorylation and nuclear translocation of transcription factor NF-κB [6], and ultimately, up-regulation of inflammatory cytokines, and chemokines that precipitate bacterial septic shock [3]. Therefore, understanding how TLR4 activation can be modulated would provide new opportunities to develop effective therapeutics for inflammatory diseases.

Herbal products have been used in traditional medicines for a wide range of infectious and inflammatory diseases [7]. Polysaccharides from plants have been shown to modify host responses and enhance immunity [8-10], but few showed immunomodulatory effects [11]. *Radix Bupleuri* (family *Umbelliferae*), known as ‘Chai-Hu’, is one of the most frequently prescribed herbs in traditional Chinese medicine for inflammatory [12] and autoimmune diseases [13]. *Bupleurum smithii* var. *parvifolium* is abundantly distributed in the northwest region of China and its roots are also used as *Radix Bupleuri* [14]. Our previous studies have confirmed that *Bupleurum* polysaccharides (BPs), extracted from *Bupleurum smithii* var. *parvifolium*, attenuated lupus-like syndrome (SLE) [14] and acute lung injury (ALI) in rodents [15]. In an *ex vivo* study, BPs suppressed LPS-induced release of pro-inflammatory cytokines but enhanced phagocytic activity of macrophages [16], suggesting that BPs can modulate cellular immune reaction to pathogens. 

Because LPS specifically binds to TLR4 and BPs can inhibit LPS, it is hypothesized that BPs exerts anti-inflammatory effects through regulating TLR4-mediated signaling. In this study, the mechanisms underlying the actions of BPs in LPS-stimulated TLR4 signaling were explored in an *in vitro* cell culture model. The activities of BPs on immunologic functions including phagocytosis and secretion of cytokines in non-LPS stimulated macrophages were also examined. To further delineate the therapeutic potential and elucidate the anti-inflammatory mechanisms, beneficial effects of BPs were tested in an *in vivo* rodent model of ALI.

## Materials and Methods

### Reagents

Cell culture medium (RPMI-1640) and endotoxin-free fetal bovine serum (FBS) were purchased from Gibco (Grand Island, NY, USA). Lipopolysaccharide from *Escherichia coli* serotype 055: B5 and Polymyxin B (PMB) were obtained from Sigma Chemical Co (St. Louis, MO, USA). *Escherichia coli* (K-12 strain)-FITC fluorescent BioParticles was from Invitrogen (Carlsbad, CA, USA). Antibodies against TLR4, CD14, and fluorochrome (FITC or PE)-conjugated antibodies were from Santa Cruz Biotechnology (Santa Cruz, CA, USA). Antibodies against TLR2, MyD88, IRAK4, TRAF6, β-actin, Histone H3, NF-κB p65, phosphor (p)-NF-κB p65 (Ser536), p-ERK (Thr^202^/ Tyr^204^), ERK, p-JNK (Thr^183^/ Tyr^185^), JNK, p-p38 (Thr^180^/ Tyr^182^), p38 were from Abgent (San Diego, CA, USA) and Cell Signaling Technology (Beverly, MA, USA). Horseradish peroxidase (HRP)-conjugated secondary antibodies and monoclonal anti-GAPDH–peroxidase antibody were from KangChen bio-tech company (Shanghai, China). ELISA kits for cytokines were from R&D systems (Minneapolis, MN, USA). 

### Drugs

The roots of *Bupleurum smithii* var. *parvifolium* were purchased from Shanghai Hua-Yu Chinese Materia Medica Co. Ltd. A voucher specimen (DFC-CH-H2003121602) of the plant material has been deposited in the Herbarium of Materia Medica (Department of Pharmacognosy, School of Pharmacy, Fudan University, Shanghai, China). The isolation and chromatographic studies of *Bupleurum* polysaccharides (BPs) were performed as previously described [14,16]. BPs contains one major polysaccharide with several minor ones, determined by high-performance gel permeation chromatography (HPGPC) analysis. Gas chromatographic analysis was utilized to assess the monosaccharide composition of BPs and the ratio of Ara, Gal, Glc, and Rha is 6.35: 3.15: 1.47: 1, along with trace of Man and Xyl. [16]. Using *Limulus amebocyte lysate* reagent method [17], the endotoxin content of BPs was determined to be 28 ppm. Prednisolone acetate was obtained from Amresco (OH, USA).

### Animals

Male BALB/c mice (6-8 weeks old) and Wistar rats (280-300 g) were purchased from Slaccas-Shanghai Lab Animal Ltd. (SPFⅡCertificate; No. SCXK 2007-0005), and housed in pathogen free condition with a free access to food and water. All experimental protocols described in this study were approved by the Animal Ethical Committee of School of Pharmacy, Fudan University (approval identification: 2011-1).

### Isolation and culture of peritoneal macrophages

Peritoneal macrophages were obtained from mice 4 days after intraperitoneal administration of 1 ml of 5% sodium thioglycollate medium [16]. Cells were harvested by flushing the cavity with 5 ml of chilled serum-free RPMI-1640 medium and the lavage was centrifuged at 80× *g* for 10 min at 4°C. Then cells were cultured in RPMI-1640 medium containing 10% heat-inactivated FBS, 100 U/ml penicillin, and 100 μg/ml streptomycin at 37°C in a humidified atmosphere with 5% CO_2_ for 2 h. Non-adherent cells were removed by washing three times with warm RPMI-1640. The adherent cells were peritoneal macrophages and used for experiments. This entire procedure was carried out in aseptic conditions and all materials were previously sterilized and pyrogen-free. The purity of macrophage preparations was more than 90%, evaluated based on cell morphology and α-naphthylacetate esterase staining. 

The macrophages were plated in tissue culture plates or dishes and treated with LPS (1 μg/ml) to stimulate inflammatory reaction. BPs (10, 20, 40, 80 μg/ml) were added to the cultures to test anti-inflammatory effect.

### Cell viability assay

 The cytotoxicity was evaluated by the 3-(4,5-dimethylthiazol-2yl)-2,5,- diphenyltetrazolium bromide (MTT) assay [18]. Briefly, cells (2×10^5^/well) were incubated with BPs (1-320 μg/ml) for 24 h in a 96-well plate. Thereafter, MTT (Sigma) was added to cell cultures to reach a final concentration of 0.5 mg/ml. After incubation for 4 h, the formazan crystals formed by the action of mitochondrial enzymes in viable cells were solubilized in dimethyl sulfoxide. The absorbance of each well was measured at 570 nm using a microplate reader (BioTek Instruments).

### Immunocytochemistry

Macrophages (1×10^6^ cells/ml) were cultured on glass coverslips (Thermo Fisher Scientific) placed in 24-well plates. After treatment with LPS and BPs for 3 h, the cells were fixed with 4 % paraformaldehyde for 20 min at room temperature and incubated with antibodies recognizing TLR4/CD14 (1:50) at 4°C overnight. After being incubated with FITC/PE-conjugated secondary antibodies (1:200) for 1 h at 37°C, the cells were counterstained with DAPI and observed under a fluorescent microscope (Carl Zeiss Inc.). For the assay of NF-κB p65 nuclear translocation, macrophages were stimulated with LPS and BPs for 1 h. The cells were then fixed with 4 % paraformaldehyde and permeabilized with 0.2% Triton X-100. Then cells were stained with rabbit anti-NF-κB p65 antibody (1:50) and incubated with Alexa Fluor 488-conjugated goat anti-rabbit IgG (1:200, Invitrogen) and propidium iodide (PI). Images were visualized by confocal microscopy (Zeiss LSM 710).

### Preparation of cell lysates and Western blot assay

Macrophages were seeded at a density of 8×10^6^ cells/dish in 60-mm plastic dishes. Cells were incubated with LPS and BPs for 24 h (for analysis of TLR4, CD14, TLR2, and MyD88) or 15 min (for analysis of IRAK4, TRAF6, NF-κB p65, p-NF-κB p65, ERK, p-ERK, JNK, p-JNK, p38, and p-p38), and harvested and incubated in lysis buffer (Beyotime Institute of Biotechnology, Shanghai, China) on ice for 20 min. The cell lysates were centrifuged at 12000× *g* at 4°C for 10 min. The supernatants were collected and mixed with 1/4 volume of 5× SDS loading buffer. To evaluate the nuclear translocation of NF-κB p65, cells were treated with LPS and BPs for 1 h. Nuclear and cytoplasmic proteins of cells were extracted using NE-PER^®^ Nuclear and cytoplasmic Extraction Reagents (Thermo Scientific) according to the manufacturer’s instructions. 

Proteins in the samples were resolved in 10% SDS-polyacrylamide gel by electrophoresis and blotted onto polyvinylidene difluoride (PVDF) immunobilon membranes (Millipore, Bedford, MA, USA). After being blocked in TBS-T solution (20 mM Tris-HCl, 137 mM NaCl, 0.1 % Tween-20, pH 7.5) containing 5% skim milk, the membranes were incubated overnight at 4°C with antibodies against TLR4, CD14, TLR2, MyD88, IRAK4 (all 1:100); TRAF6, β-actin, Histone H3, NF-κB p65, p-NF-κB p65, ERK, p-ERK, JNK, p-JNK, p38, p-p38 (all 1:1000). After incubation with HRP-conjugated secondary antibodies for 1 h at room temperature, signals were detected by enhanced electrochemiluminescence (ECL) reagent and captured with a camera-based imaging system (Alpha Innotech, Santa Clara, CA, USA). Density of the signals was quantified using AlphaEase software.

### ELISA and Griess assay

Macrophages (1×10^6^ cells/ml) were treated with LPS and BPs for 24 h, and the culture media was collected to assess the levels of TNF-α, IL-6, IL-1β, IL-12p40, IL-10, and IFN-β using enzyme-linked immunosorbent assay (ELISA) kits (R&D systems), following the manufacturer’s introductions.

NO production in the cells treated with LPS and BPs for 24 h was tested using the Griess reagent to detect its end product, nitrite [19]. Briefly, the culture media (100 μl) was mixed with 100 μl Griess reagent [mixture of equal volume of 0.1% N-(1-naphthyl) ethylenediamine dihydrochloride in H_2_O and 1% sulfanilamide in 5% H_3_PO_4_] in a 96-well plate and incubated at room temperature for 15 min. The optical density was measured using a spectrophotometer at a wave length of 540 nm. The concentration of nitrite was determined with reference to a standard curve generated with sodium nitrite.

### Phagocytosis assay

The phagocytosis was assayed by detecting ingestion of *E.coli* fluorescent particles by macrophages [20]. Macrophages (2×10^5^ cells/ml) were placed in 24-well culture plates and treated with different concentrations of BPs for 30 min. A suspension of *E.coli*-FITC fluorescent bioparticles in culture medium (3×10^8^ *E*.*coli*/ml, 10 μl) was added to the cell cultures (*E.coli* particles/cells =15:1). After incubation for 1 h, non-engulfed particles were washed away with PBS. Then the cells were fixed with 4% paraformaldehyde and observed under a fluorescent microscope. At least 200 macrophages were counted. The value of phagocytosis was calculated as a percentage of cells with internalized fluorescent bioparticles in a total number of cells. The intracellular fluorescence intensity was measured by Varioskan Flash (Thermo Scientific).

### Rat model of ALI

Rats were randomly divided into five groups: sham group, model group, BPs (5, 10 mg/kg) and prednisolone acetate (70 mg/kg) treated group. BPs was dissolved in normal saline for oral administration. Sham and model group were given normal saline in the same route. Prednisolone acetate, used as the positive control, was injected intraperitoneally. The animals were anaesthetized with an intraperitoneal injection of 1 g/kg of urethane. The right carotid artery was cannulated for monitoring the mean arterial pressure (MAP), blood sampling, and resuscitation. Hemorrhagic shock was initiated by blood withdrawal, which reduced MAP to 45 ± 5 mm-Hg within 20 min. This blood pressure was maintained by additional blood withdrawal when MAP was >50 mm-Hg, and by infusion of 0.2 ml of saline when MAP was < 40 mm-Hg. Blood samples were collected and 4 % heparin was added to prevent clotting. After being hypotensive for 1 h, rats were resuscitated by transfusion of shed blood for another 1 h. 

Drugs were administered 30 min after the starting of resuscitation. At the end of resuscitation, saline (sham group) or 0.2 mg/kg LPS were administered to the lungs via intratracheal instillation through a tracheotomy tube [21]. During the course after intratracheal instillation, blood was collected for measurement of CO_2_ concentration, which was determined using commercial kits (Nanjing Jiancheng Bioengineering Institute, Nanjing, China).

2.5 h after saline or LPS instillation, the rats were exsanguinated and sacrificed. The lungs were removed and fixed in 10% formalin and subsequently embedded in paraffin wax. Tissue sections in 5 μm thickness were stained with hematoxylin and eosin (HE) and observed under a microscope. 

### Immunohistochemistry

Lung sections were dewaxed, rehydrated, and heated in 10mM citrate buffer (pH 6.0) for 20 min to retrieve antigens. After quenching endogenous peroxidase with 3 % hydrogen peroxide and blocking non-specific binding with 5% BSA, the sections were incubated at 4°C overnight with murine monoclonal anti-TLR4 antibody (1:100, Abcam), followed by incubation with HRP-conjugated goat anti-mouse IgG antibody. Signals were visualized using 3-3’ diaminobenzidine (DAB) substrate-chromogen solution. The sections were then counterstained with hematoxylin and observed under a microscope. Images were acquired using an image analysis system.

### Statistical analysis

Data were presented as mean ± standard deviation (SD). Statistical comparisons between multiple groups were performed using one-way ANOVA, followed by Dunnet’s post-hoc test. *P* value of 0.05 or less was considered statistically significant. 

## Results

### Evaluation of BPs cytotoxicity in macrophages

Exposure of macrophages to 1-80 μg/ml BPs for 24 h showed no significant increases in cell death (Figure S1), indicating that BPs at the concentrations used in this study were not cytotoxic.

### Suppression of LPS-stimulated TLR4 and CD14 expression by BPs in macrophages

**Figure 1:**
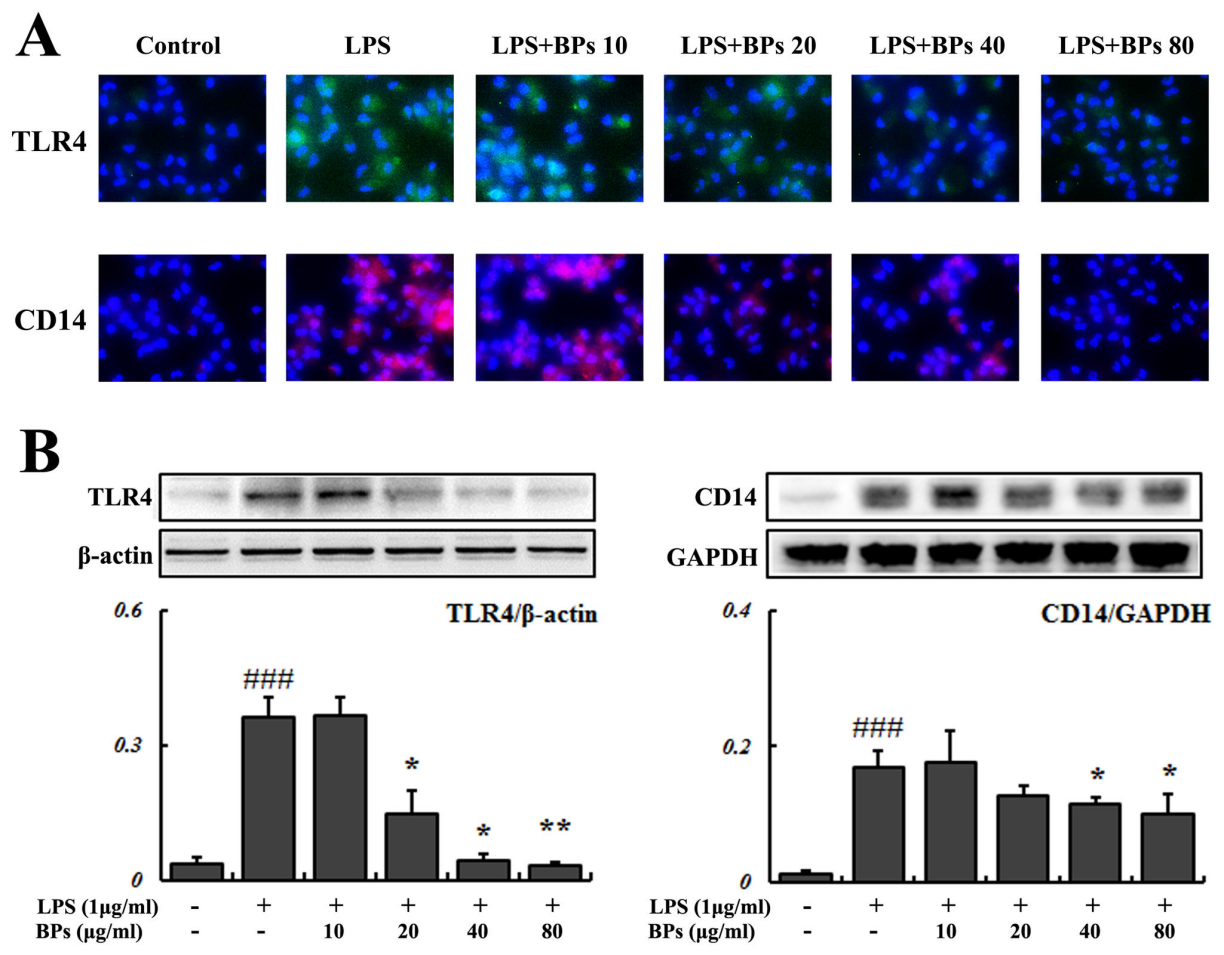
Effects of BPs on LPS-induced TLR4 and CD14 expression. Macrophages were treated with medium only, LPS (1 μg/ml), or LPS in the presence of increasing concentrations of BPs (10-80 μg/ml). (A) Immunocytochemistry of TLR4 (green color) and CD14 (red color) in macrophages treated for 3 h. (B) Western blot quantification of TLR4 and CD14 after 24 h of treatment. β-actin and GAPDH are loading controls. Data are presented as mean ± SD and are representative of three independent experiments. ^###^ *P*<0.001 compared with control group; ^*^ *P*<0.05, ^**^ *P*<0.01 compared with LPS group.

Binding of LPS to CD14 and the subsequent formation of a complex with TLR4 initiate the LPS-TLR4 signaling [5]. As shown in Figure 1A, non-LPS stimulated cells exhibited weak staining, suggesting a low basal level of TLR4 and CD14. After LPS stimulation, dramatic increases were seen in TLR4 and CD14 expression, assessed by immunocytochemistry. By comparison, such elevations were blunted by BPs treatment. Quantification of these changes using Western blot assay showed that LPS caused a marked increase in TLR4 and CD14 expression, compared with control (*P*<0.001; Figure 1B); and BPs (40, 80 μg/ml) significantly inhibited the effects of LPS (*P*<0.05; Figure 1B).

### Suppression of LPS-stimulated TLR2, IRAK4, and TRAF6 expression by BPs in macrophages

**Figure 2:**
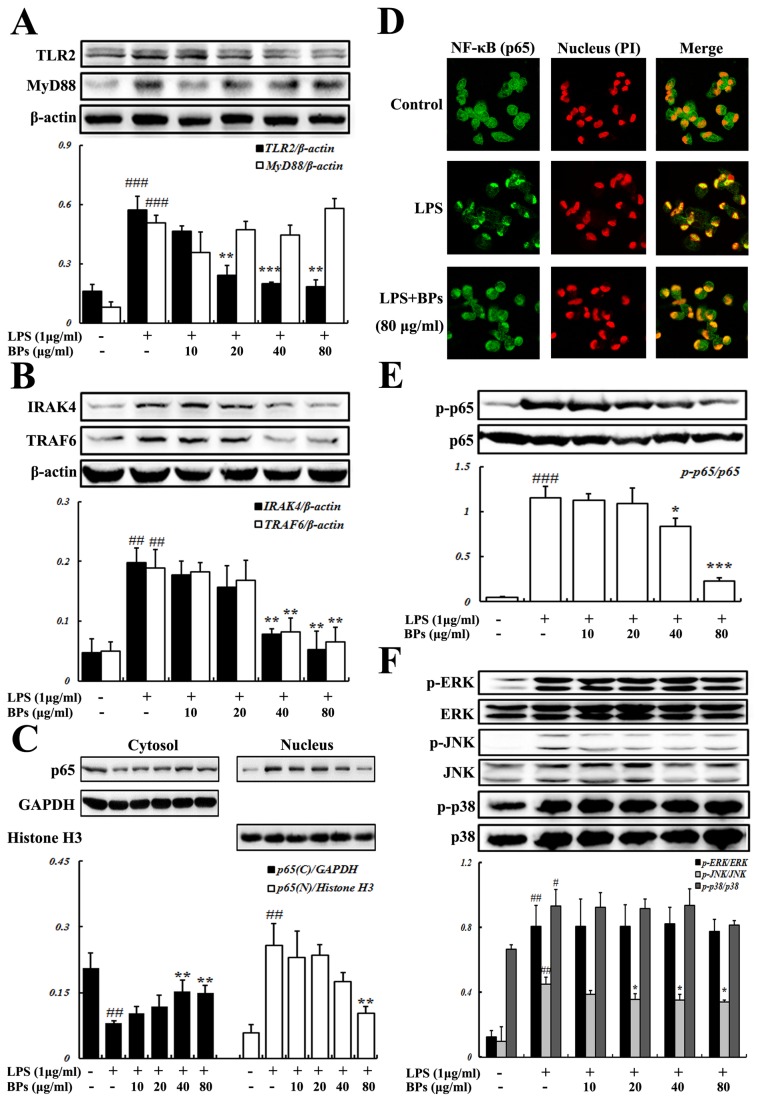
Effects of BPs on LPS-induced activation of factors in the TLR4 signaling system. Macrophages were incubated with medium only, LPS, or LPS in the presence of BPs. (A) Expression of TLR2 and MyD88 after 24 h of treatment; and (B) Expression of IRAK4 and TRAF6 after 15 min of treatment were determined by Western blot analysis, β-actin is loading control. (C) Levels of NF-κB p65 subunit in the cytosol and nucleus after 1 h of treatment were analyzed by Western blot, GAPDH and Histone H3 are used as loading controls for cytosolic and nuclear proteins, respectively. (D) NF-κB p65 nuclear translocation after 1 h of treatment was detected by confocal microscopy. (E) Phosphorylation of NF-κB p65 after 15 min of treatment; and (F) Phosphorylation of ERK, JNK, p38 after 15 min of treatment were evaluated by Western blot analysis. Data are presented as mean ± SD and are representative of three independent experiments. ^#^ *P*<0.05, ^##^ *P*<0.01, ^###^ *P*<0.001 compared with control group; ^*^ *P*<0.05, ^**^ *P*<0.01, ^***^ *P*<0.001 compared with LPS group.

The LPS-TLR4 signaling involves many factors, including MyD88, IRAK4 and TRAF6. TLR2 has also been implicated in LPS-mediated signaling [22]. In macrophages stimulated with LPS, increases in the expression of TLR2 and MyD88 were observed, compared with the control (*P*<0.001; Figure 2A). In addition, treatment with LPS obviously enhanced the expression of IRAK4 and TRAF6 (*P*<0.01; Figure 2B). BPs (40, 80 μg/ml) significantly decreased LPS-induced expression of TLR2, IRAK4, and TRAF6 (*P*<0.01), but not that of MyD88 (Figure 2A, 2B). 

### Suppression of LPS-stimulated NF-κB p65 and JNK activation by BPs in macrophages

The LPS-TLR4 signaling activates members of the MAPKs family, including ERK, JNK, and p38 via phosphorylation [23], leading to phosphorylation and nuclear translocation of the transcription factor NF-κB [24]. To further investigate the mechanisms underlying the anti-inflammatory effect of BPs, activation of NF-κB p65 subunit and MAPKs was evaluated. As shown in Figure 2C, LPS dramatically decreased cytosolic p65 level but elevated its translocation into the nucleus, compared with the control (*P*<0.01). Co-treatment with BPs (80 μg/ml) and LPS significantly increased level of p65 in the cytosol and reduced its nuclear level based on immunoblot analysis (*P*<0.01). Direct observation of p65 localization after BPs treatment was done using confocal microscopy. Apparent increase of p65 in the nucleus was visualized in LPS-stimulated cells in contrast to that in control group, and BPs (80 μg/ml) inhibited LPS-induced p65 nuclear translocation (Figure 2D). To further examine the role of BPs in regulation of NF-κB, the phosphorylation of p65 was examined. As displayed in Figure 2E, after cells were stimulated with LPS, significantly induced phosphorylation of NF-κB p65 was observed (*P*<0.001). However, BPs (40, 80 μg/ml) strongly blunted LPS-stimulated p65 phosphorylation (*P*<0.05). 

LPS increased the phosphorylation of ERK, JNK, and p38, compared with the control (*P*<0.05). BPs (20, 40, 80 μg/ml) potently suppressed LPS-stimulated JNK phosphorylation (*P*<0.05), but not ERK and p38 (Figure 2F).

### Suppression of pro-inflammatory cytokines and NO production by BPs in LPS-stimulated macrophages

**Figure 3:**
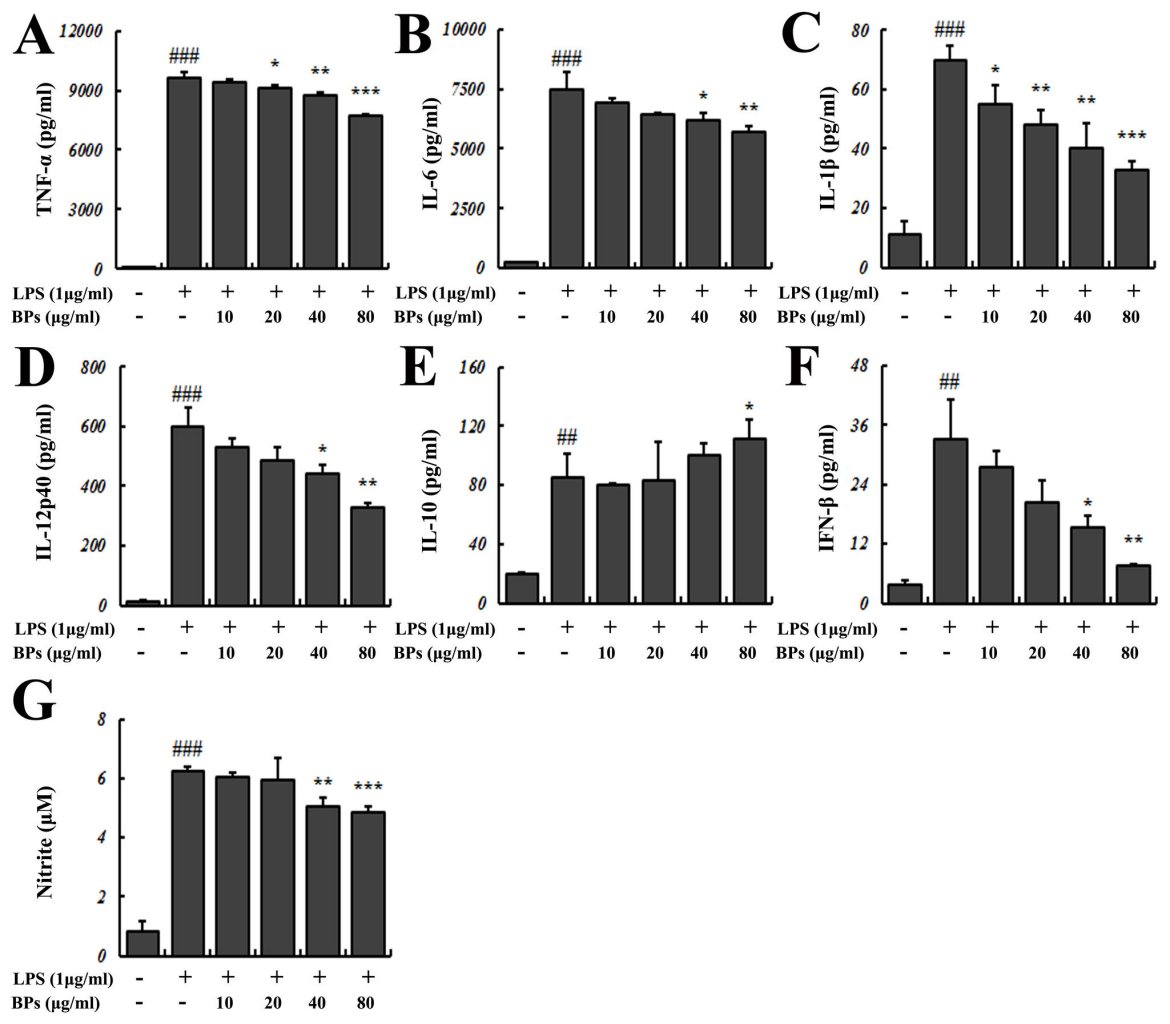
Effects of BPs on LPS-induced inflammatory cytokines and NO production. Macrophages were stimulated for 24 h with medium only, LPS, or LPS in the presence of BPs. Supernatants were analyzed for (A) TNF-α, (B) IL-6, (C) IL-1β, (D) IL-12p40, (E) IL-10, (F) IFN-β, and (G) NO production by ELISA and Griess reaction assays. Data are presented as mean ± SD and are representative of three independent experiments. ^##^ *P*<0.01, ^###^ *P*<0.001 compared with control group; ^*^ *P*<0.05, ^**^ *P*<0.01, ^***^ *P*<0.001 compared with LPS group.

Activation of NF-κB, as a process of inflammatory response, results in up-regulation of pro-inflammatory cytokines and iNOS/NO [3]. Treatment with LPS significantly increased the levels of pro-inflammatory mediators, including TNF-α, IL-6, IL-1β, IL-12p40, IFN-β, and NO (*P*<0.01; Figure 3). In contrast, BPs (40, 80 μg/ml) significantly decreased LPS-induced augmentation of these factors (*P*<0.05; Figure 3). 

The level of IL-10, an anti-inflammatory and immunosuppressive cytokine, was elevated by LPS (*P*<0.01; Figure 3E). BPs (80 μg/ml) further promoted the IL-10 production in LPS-stimulated cells (*P*<0.05; Figure 3E). 

### Increases in pro-inflammatory cytokines production by BPs

**Figure 4:**
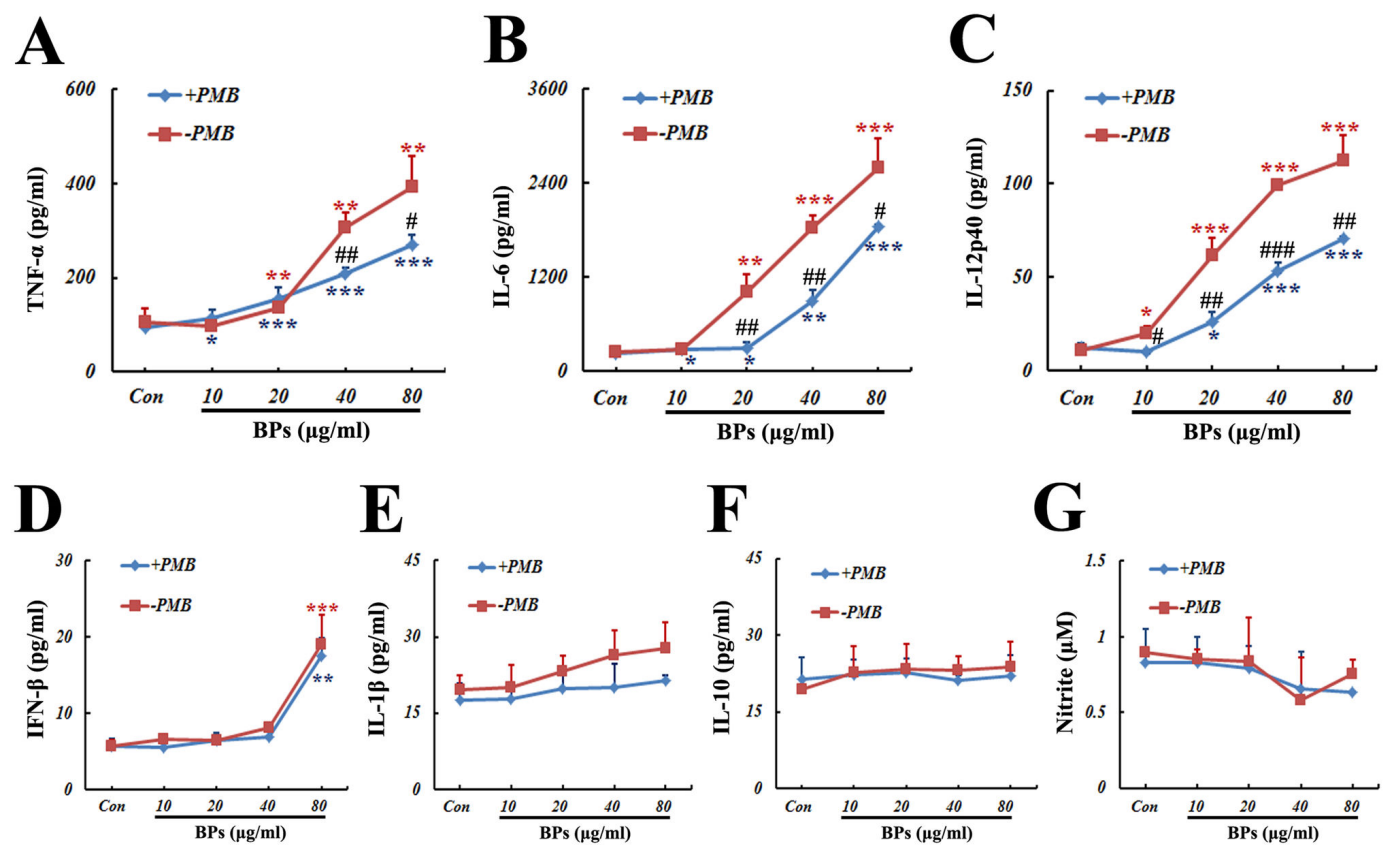
Effects of BPs on cytokines and NO secretion. Each concentration of BPs was pretreated with (blue line) or without (red line) PMB (1 μg/ml) at 37 °C for 24 h, and then added to the cell cultures. After macrophages were treated for 24 h the culture media were harvested and assayed for (A) TNF-α, (B) IL-6, (C) IL-12p40, (D) IFN-β, (E) IL-1β, (F) IL-10, and (G) NO. Data are presented as mean ± SD and are representative of three independent experiments. ^*^ *P*<0.05, ^**^ *P*<0.01, ^***^ *P*<0.001 compared with corresponding control group; ^**#**^ *P*<0.05, ^**##**^ *P*<0.01, ^**###**^ *P*<0.001 compared between groups with and without PMB.

To determine whether BPs itself can affect the immunologic functions of macrophages, production of cytokines and NO was assessed in the cells treated with BPs alone. Substantial increases in TNF-α, IL-6, IL-12p40, and IFN-β were observed (*P*<0.05; Figure 4A, 4B, 4C, 4D). However, at the doses used, BPs treatment failed to influence the secretion of IL-1β, IL-10, and NO (Figure 4E, 4F, 4G).

To exclude the possibility that the results above were attributed to the contamination of bacterial endotoxin in BPs, BPs was pretreated with polymyxin B (PMB), a well-characterized LPS inhibitor [25] at 37°C for 24 h, and, then, added to the cell cultures. It has been reported that 3 μg/ml PMB is sufficient to inhibit up to 10 ng/ml LPS [26]. Thus, 1 μg/ml PMB was used in blocking the approximate 2.24 ng/ml LPS in 80 μg/ml BPs. As seen in Figure 4, significantly lower levels of TNF-α, IL-6, and IL-12p40 were observed in the BPs+PMB treatment group, compared with the BPs group (P<0.05). No differences were seen for IFN-β, IL-1β, IL-10, and NO. 

### Enhanced phagocytosis of macrophages by BPs

**Figure 5:**
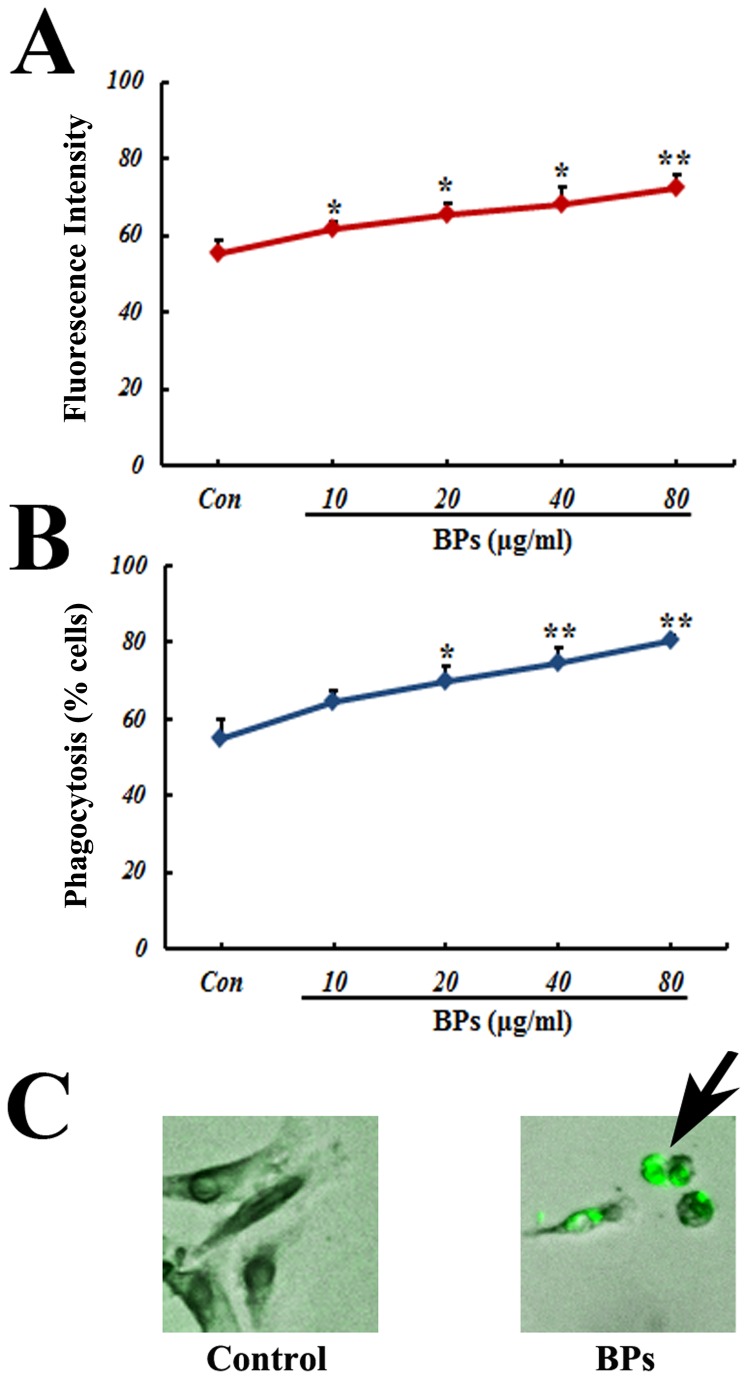
Effect of BPs on phagocytosis of *E.coli*. Macrophages were either cultured in medium only or pretreated for 30 min with BPs. Phagocytosis was assayed using fluorescent *E.coli* bioparticles. (A) Intracellular fluorescence intensity; (B) Rate of phagocytosis; and (C) Microscopic representation of phagocytosis. Arrow indicates the phagocytosed *E.coli* particles. Data are presented as mean ± SD and are representative of six independent experiments. ^*^ *P*<0.05, ^**^ *P*<0.01 compared with control group.

Preincubation of cells with BPs resulted in a dose-dependent increase of phagocytic activity towards *E.coli* bioparticles. The intracellular fluorescence intensity was notably higher in the BPs-treated groups (10-80 μg/ml) than that in the control group (*P*<0.05; Figure 5A, 5C). And the number of cells containing *E.coli* bioparticles was distinctly larger in BPs-treated groups (20-80 μg/ml) than that in the control group (*P*<0.05; Figure 5B).

### Alleviation of inflammation by BPs in ALI model of rats

**Figure 6:**
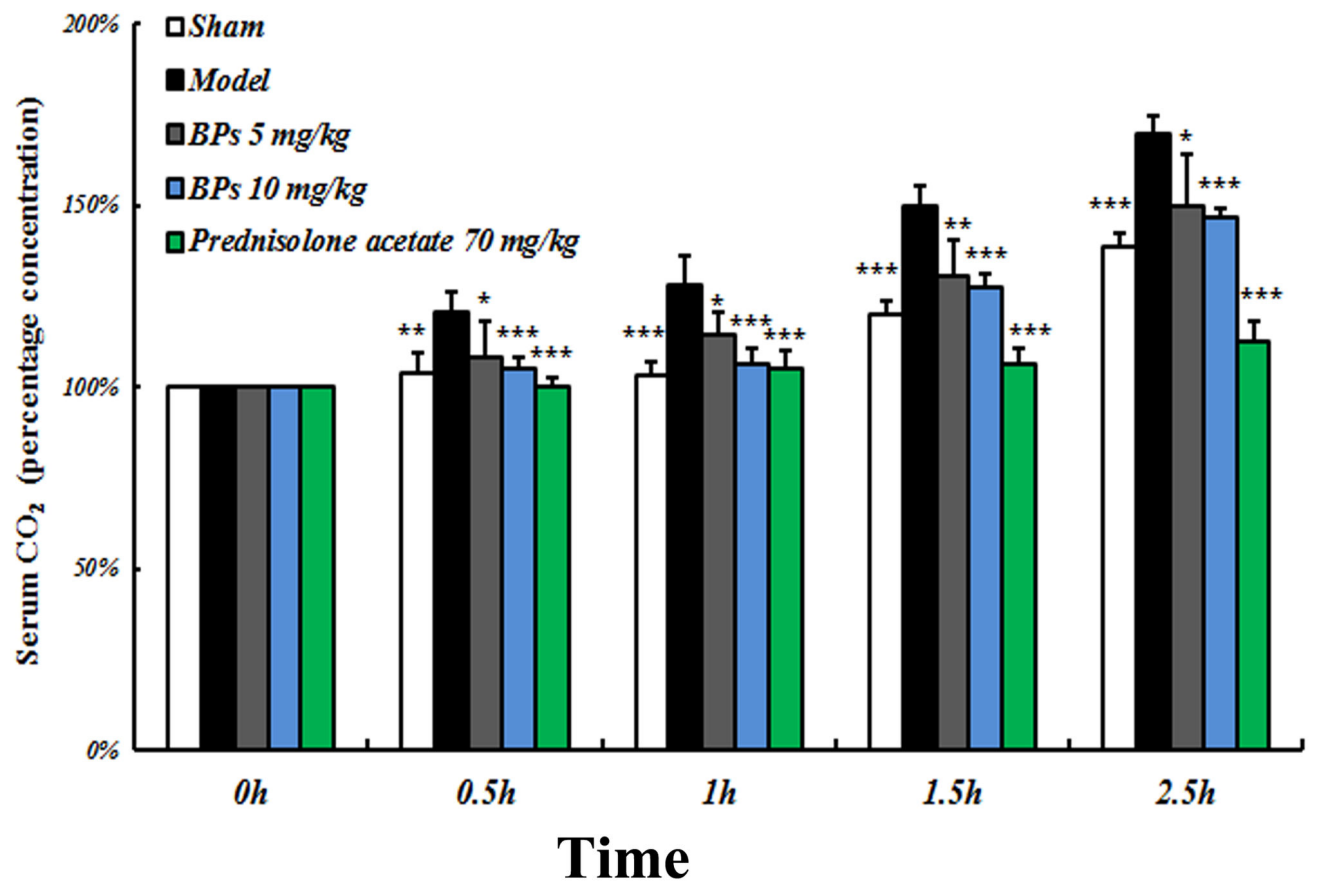
Effect of BPs on serum CO_2_ concentration of hemorrhagic shock and LPS induced ALI rats. Rats were administrated with BPs (5, 10 mg/kg) or prednisone acetate (70 mg/kg) and then received intratracheal instillation of normal saline or LPS (0.2 mg/kg) for the indicated time periods. At each time point the blood was collected and serum concentration of CO_2_ was tested. Results are presented as mean ± SD and are representative of eight independent experiments. ^*^ *P*<0.05, ^**^ *P*<0.01, ^***^ *P*<0.001 compared with model group.

To examine the anti-inflammatory effect of BPs *in vivo* and to further substantiate the inhibitory efficiency of BPs on LPS-mediated TLR4 activation, an animal model of ALI was developed. As shown in Figure 6, striking increases in the level of serum CO_2_ were detected 0.5 h to 2.5 h after LPS administration in contrast with sham group (*P*<0.01). Treatments with BPs (5, 10 mg/kg) or prednisolone acetate (70 mg/kg) markedly reduced the concentration of CO_2_ as compared with model group (*P*<0.05). 

**Figure 7:**
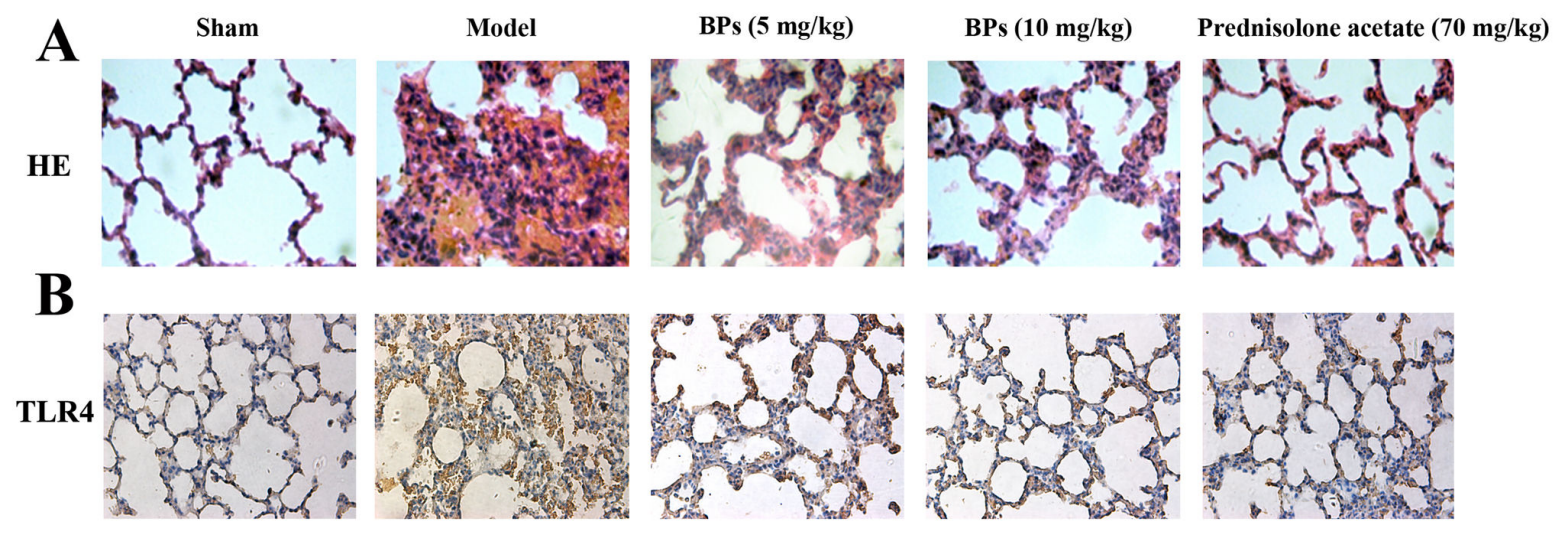
Effects of BPs on amelioration of lung injuries and attenuation of TLR4 expression in ALI rats. (A) Histopathology findings and (B) TLR4 expression of the lungs were detected from sham, model, BPs or prednisolone acetate treated groups 2.5 h after inhalation of saline or LPS, as determined with HE staining and immunohistochemistry analysis. The representative photomicrographs are chosen from one of eight independent experiments which yielded similar results.

Histopathological examination of the lung sections of ALI rats revealed severe pulmonary inflammation, characterized with neutrophilic infiltration, oedema, parenchymal hemorrhage, atelectasis, and alveolar atrophy (Figure 7A). Nevertheless, few signs of pathological changes were found in the sham group. Treatments with BPs or prednisolone acetate obviously ameliorated the lung injuries, which demonstrated a reduction in the severity of pulmonary inflammation (Figure 7A). 

Immunohistochemistry showed faint positive staining for TLR4 in the lungs of sham group. In contrast, strong positive expression of TLR4 was visualized around the alveolar epithelial cells in the lungs of ALI rats. In BPs and prednisolone acetate treatment groups, the intensities and densities of TLR4-positive staining were weak and sparse (Figure 7B). 

## Discussion

As a traditional Chinese herbal medicine, *Bupleurum* has been extensively used to treat various inflammatory diseases [27]. Although BPs has been reported to possess anti-inflammatory properties *in vivo*, the molecular mechanisms responsible for these actions remain unclear. Considering tissue macrophages play pivotal roles in the pathogenesis of multiple LPS-induced inflammatory diseases, it is essential that drugs used to ameliorate these diseases should act effectively on macrophages. Herein, we have demonstrated, for the first time, that BPs attenuates LPS-induced macrophage inflammatory responses via modulating TLR4 signal transduction. The effects of BPs include suppressing expression of TLR4, CD14, TLR2, IRAK4, and TRAF6; inhibiting activation of NF-κB and JNK; and reducing production of pro-inflammatory cytokines and NO. In addition, BPs alone can up-regulate the phagocytic activity of macrophages to eliminate bacterial pathogens, and augment the generation of certain pro-inflammatory cytokines, indicating that BPs is capable to enhance immunologic functions of macrophage in non-inflammatory conditions. More importantly, BPs protects the lungs against hemorrhagic shock and LPS-induced ALI and inhibits TLR4 expression *in vivo*. All these findings indicate that BPs exerts beneficial effects on inflammatory diseases through inhibiting LPS-induced TLR4 signaling. 

TLR4 and CD14 are recognized as the apex of cellular responses to LPS and as targets for drug development strategies [28]. BPs can attenuate the inflammatory reactions induced by LPS, which is probably associated with significant suppression of TLR4 and CD14 expression. It can be inferred that BPs possesses an ability to disrupt the interaction of LPS with TLR4/CD14 and targets upstream event in LPS-mediated signaling. 

In addition to TLR4, TLR2 can also be induced by LPS, although it is not a physiological receptor for LPS. TLR2 specifically recognizes components from Gram-positive bacteria with the assistance of scavenger receptor CD36 [29]. BPs also blocks LPS-induced up-regulation of TLR2 in macrophages, demonstrating that BPs may affect a large spectrum of bacterial infection. Upon activation by LPS, TLR4 forms homodimers and recruits adaptor protein MyD88, which subsequently interacts with IRAK4 and TRAF6, leading to activation of downstream mediators such as NF-κB [4]. BPs suppresses LPS-induced expression of IRAK4 and TRAF6, but not MyD88. It is possible that because MyD88 is required for the signaling of nearly all TLRs [4], and its expression is controlled by multiple signaling systems, in addition to that of TLR4. In light of this notion, it is also possible that BPs acts selectively through the LPS signaling to suppress the MyD88 expression, while other systems may augment its expression as compensatory regulation. Further investigations are needed to address the mechanisms. Nevertheless, our data suggest that BPs acts on the LPS-induced signaling progress after the activation of TLR4.

NF-κB is an important transcription factor in the LPS-TLR4 signaling to control the expression of numerous inflammatory genes, such as iNOS, TNF-α, IL-1β, IL-6, and IL-12 [24]. The activation of MAPKs is critical in mediating a broad array of cellular responses, including cell proliferation and differentiation, transcription factor activation, cytokine gene expression and production [23]. Thus, inhibitors of NF-κB and MAPKs have been used as therapeutic drugs in clinical applications for inflammation-associated human diseases [30]. Here, we show that BPs potently suppresses LPS-induced NF-κB p65 activation. In addition to p65 phosphorylation, elevation of p65 level in cytosol and reduction of it in nucleus is also observed upon BPs treatment, suggesting that BPs blocks the mobilization of p65 into nucleus and attenuates NF-κB-mediated signaling. Moreover, BPs inhibits LPS-induced JNK phosphorylation. It was reported that both inhibited activation of NF-κB and JNK result in decreases in the expression of cytokines, including TNF-α, IL-6, IL-1β, and IL-12 [31]. And NF-κB as well as MAPKs is involved in the signaling pathway for LPS-induced iNOS expression, which produces NO, an essential factor in inflammation [23,32]. Thus, the inhibitory property of BPs on LPS-induced generation of pro-inflammatory cytokines and NO is probably associated with the attenuated NF-κB and JNK activation. All these findings exhibit a profoundly inhibitory role of BPs in the MyD88-dependent pathway of LPS-TLR4 signal transduction since pro-inflammatory cytokines and NO are major products downstream of MyD88. 

Unlike pro-inflammatory cytokines, IL-10 acts to limit excessive generation of pro-inflammatory cytokines by decreasing cytokine gene transcription and regulating the stability or translation of target mRNAs [33]. Our results show that IL-10 expression is elevated by BPs, implicating a novel mechanism underlying the action of BPs in inflammatory modulation. IFN-β is a critical inducer of endotoxic shock following LPS exposure, and expression of IFN-β relies on activation of the TRIF-dependent pathway in the LPS-TLR4 signaling system [34]. Our data show that BPs inhibits LPS-induced IFN-β expression, indicating that BPs may also target the TRIF-dependent pathway to mediate inflammatory responses. 

Interestingly, BPs alone can stimulate the expression of pro-inflammatory cytokines, including TNF-α, IL-6, IL-12p40, and IFN-β, in normal macrophages. Whereas, compounds such as polysaccharides obtained from natural sources can easily be contaminated with LPS, since bacteria are ubiquitous organisms and interfere with biological evaluation experiments [35]. To establish that BPs-induced cytokines release could not be accounted for LPS contamination, BPs is pretreated with PMB, a drug which binds to charged regions of LPS to prevent cell activation by LPS [36]. In line with the previous studies indicating that TNF-α, IL-6, and IL-12p40 are particularly sensitive to trace amounts of LPS [26,37], treatment with PMB reduces but not abolish BPs-induced production of these cytokines. However, PMB fails to change BPs-mediated IFN-β, IL-1β, IL-10, and NO secretion, suggesting these mediators might not be activated by minute quantities of LPS in BPs. Taken together, BPs-mediated production of cytokines and NO is almost LPS independent. 

At the cellular level, the major function of macrophage is phagocytosis to eliminate microbial pathogens. Our previous *ex vivo* study has shown that BPs can enhance the phagocytosis of macrophages [16]. In the present study, we also elucidate that BPs elevates the phagocytic activity towards *E.coli* of macrophages, suggesting BPs can promote bacterial clearance. This phenomenon further demonstrates that BPs can enhance the immunologic functions in non-inflammatory condition, although the specific mechanisms of BPs for elevated pro-inflammatory cytokines production and phagocytic activities via definite signal transduction tunnel remain to be addressed.

LPS has been identified as a principal component in the causation of ALI [38]. Resuscitated hemorrhagic shock is believed to promote the development of lung injury by priming the immune system for an exaggerated inflammatory response to LPS [21]. Elevated blood CO_2_, known as hypercapnia, is common in patients with lung infections and is associated with increased incidence of death from pneumonia [39]. Using a rat model of ALI, we have shown in this study that BPs strikingly reduces serum CO_2_ level and ameliorates lung damages, revealing that BPs is a potential therapeutic agent for lung injuries. Meanwhile, similar to the action for attenuating LPS-mediated TLR4 signaling in macrophages, BPs decreases the expression of TLR4 in the lungs of ALI rats. It has been reported that TLR4 is required for both hemorrhage- and LPS-induced lung injury [40]. Thus, targeting TLR4 appears to be an approach to tackle lung diseases from multiple causes. BPs is promising candidate for this type of therapy.

In conclusion, we have demonstrated that BPs attenuates LPS-stimulated inflammatory reaction by regulating TLR4 signal transduction in macrophages. The amelioration of BPs on hemorrhagic shock and LPS induced lung injuries could be accounted for its inhibitory potential on TLR4 signaling. Therefore, BPs may offer a new therapeutic approach for inflammatory diseases. 

## Supporting Information

Figure S1 **Cytotoxic effect of BPs in macrophages.** Macrophages were treated with increasing concentrations of BPs (1-320 μg/ml) for 24 h and cell viability was assessed by MTT assay. Untreated cells were used as control of viability (100%) and results were expressed as % relative to control. Data are presented as mean ± SD and are representative of four independent experiments. ^*^ *P*<0.05, ^***^ *P*<0.001 compared with control group.
(TIF)
