# Supplementary figures and images for: Bupleurum Polysaccharides Attenuates Lipopolysaccharide-Induced Inflammation via Modulating Toll-Like Receptor 4 Signaling
Source: PLoS One. 2013 Oct 22;8(10):e78051. doi: 10.1371/journal.pone.0078051 (PMC3805517; doi:10.1371/journal.pone.0078051)

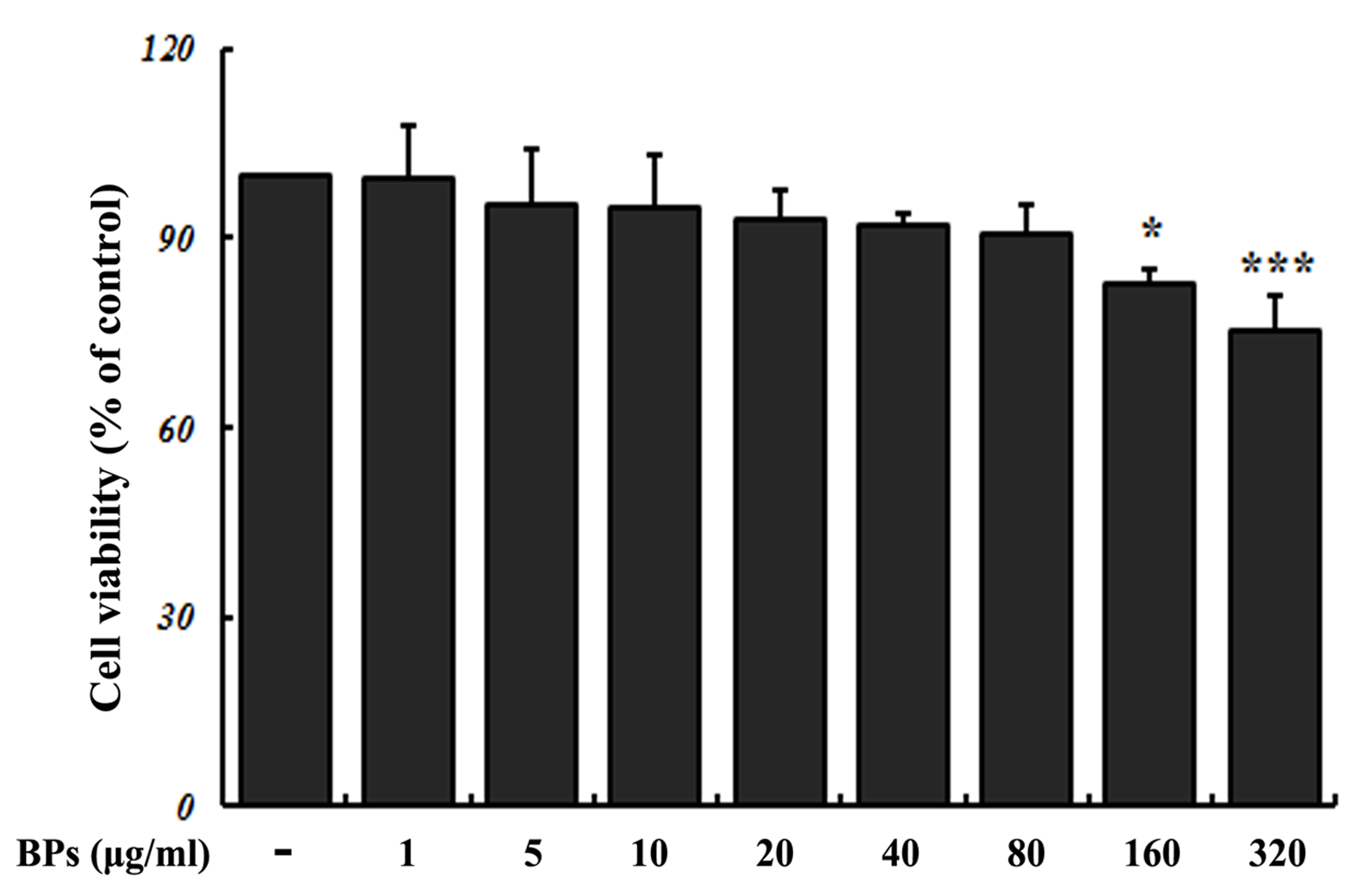

Supplement: Figure S1 — Cytotoxic effect of BPs in macrophages. Macrophages were treated with increasing concentrations of BPs (1-320 μg/ml) for 24 h and cell viability was assessed by MTT assay. Untreated cells were used as control of viability (100%) and results were expressed as % relative to control. Data are presented as mean ± SD and are representative of four independent experiments. * P<0.05, *** P<0.001 compared with control group. (TIF) [file pone.0078051.s001.tif]
